# Stop Silencing Nursing Complexity: Why Standardized Nursing Terminologies Must Be Heard

**DOI:** 10.3390/nursrep16010028

**Published:** 2026-01-16

**Authors:** Manuele Cesare, Richard Gray, Antonello Cocchieri

**Affiliations:** 1 A. Gemelli IRCCS University Hospital Foundation, 00168 Rome, Italy; antonello.cocchieri@policlinicogemelli.it; 2 Section of Hygiene, Department of Life Sciences and Public Health, Catholic University of the Sacred Heart, 00168 Rome, Italy; 3 School of Nursing and Midwifery, La Trobe University, Melbourne, VIC 3086, Australia; r.gray@latrobe.edu.au

We built a system that measures what is easy—not what matters. For decades, patient complexity has been treated as if administrative groupers, such as Diagnosis-Related Groups (DRGs), could capture it completely [1,2,3]. The result is a partial representation of clinical reality: tidy case-mix weights, elegant dashboards, and payment models that flatten the one dimension that never is—nursing. More than forty years ago, John D. Thompson warned that DRGs were never designed to reflect intensity of care, least of all nursing intensity, and that bundling nursing within “room and board” would have consequences for budgets, staffing, and safety [4,5,6].

Today, empirical evidence corroborates this concern. When standardized nursing terminologies (SNTs) are routinely recorded in electronic health records (EHRs), they illuminate clinical risk that administrative data alone leave underrepresented. And this is not surprising, given that nursing care accounts for 40 to 60% of the direct costs of hospital care [1] yet remains largely invisible in data systems and performance metrics.

The number of nursing diagnoses (NDs) and nursing actions (NAs)—measures of nursing care complexity—increase with DRG weight—a proxy of medical complexity—and vice versa [7]. In other words, the more medically complex the patient, the greater and more resource-intensive the nursing care required. This alignment clearly demonstrates that nursing complexity mirrors medical complexity and resource use at the bedside, revealing dimensions of patient care that administrative data alone cannot capture. Importantly, this also suggests that DRG weight—commonly treated as a stable proxy for medical severity—may offer only a partial representation of the underlying clinical picture. Patients who appear similar through administrative codes can differ substantially in physiological instability, functional status, and care needs.

Moreover, ND loads on admission move in lockstep with outcomes we swear we care about, including length of stay (LOS), intra-hospital transfers, and odds of later ICU transfer [8]. High nursing complexity is not a soft “signal”—it multiplies the odds of ICU transfer by a factor that no risk committee could dismiss [9]. These are not isolated findings. They are consistent, replicable patterns observed across settings and over time.

And what about LOS? The story usually follows a familiar script: researchers adjust for age, comorbidities, and a maze of case-mix variables, and then attribute the remaining unexplained variation—the residuals—to chance. Yet when standardized nursing data are included, that “unexplained” portion shrinks dramatically. The number of NDs documented on admission independently predict both LOS and prolonged LOS, even after controlling for all conventional factors [10]. In large patient cohorts, NAs are not only associated with LOS—they are its strongest independent predictor and partly mediate how medical diagnoses translate into longer hospital stays [11]. In simple terms: what nurses document and do is not just a response to complexity—it actively shapes outcomes. Years ago, a systematic review already reached the same conclusion: NDs predict both patient and organizational outcomes and improve DRG-based models [12]. Despite this, systematic integration into routine analytics has remained limited.

These findings also prompt a broader reflection: if nursing data can reveal clinical risk that administrative systems were assumed to represent, this may indicate that even medical complexity is only partially captured by DRG weight. The underlying clinical condition appears more nuanced than what billing codes can describe.

If SNTs carry this much signal, their limited use today represents a missed opportunity rather than a deliberate exclusion. Our information architecture still relegates nursing to narrative silos, while incentives reward what DRGs can already see. As a result, resource planning and risk monitoring may underrepresent the true care demands of hospitalized patients.

The fix is not a moonshot. It is a practical shift: structure the data, standardize its capture, and make it count. In EHRs, SNT fields must be first-class citizens—coded, time-stamped, and exportable—so ND/NA data are no longer buried in prose. At the hospital level, nursing complexity should share dashboards with DRG case-mix, not as a footnote but as a co-equal signal for staffing and safety [7,9]. At the journal level, we need a “Nursing Data Statement” as a non-negotiable as trial preregistration: were NDs/NAs collected, when were they collected, how were they mapped, how were they used, and with what associations?

Consider the consequences of inaction. Payment models that ignore nursing complexity may divert resources from the wards that need them most. Risk-adjustment methods that exclude NDs and NAs risk embedding systematic bias into quality assessment, outcome evaluation, performance metrics, and reimbursement systems. Predictive tools without nursing data risk are nothing more than automated hindsight. Evidence now shows what happens when we actually examine SNTs such NDs and NAs: they flag who is likely to bounce across wards, who will spiral to ICU, and who will need more time to stabilize [7,9,13]. Across multiple studies, ND loads at admission are a red siren for longer LOS and higher resource absorption [8,10,14]. In a large cohort with one-year follow-up, the combination of high nursing complexity and poor health literacy marked the worst survival and the heaviest acute-care use—exactly the group we claim our healthcare systems are built to protect [15]. There is also an ethical imperative. We celebrate nursing as the most trusted profession [16,17], yet our data systems mute the very contributions that trust is built on. Nurses track risk at the bedside with a granularity that administrative codes cannot approach. When standardized, this information does more than describe care—it predicts what happens next [13]. Ensuring that this predictive knowledge informs decisions is consistent with principles of fairness and responsible resource allocation.

We do not need perfect ontologies or multinational consortia to start. We need line-of-sight from bedside documentation to unit decisions, and from those decisions to the models and money that shape care. Hospitals can map existing ND/NA fields, display them alongside DRG case-mix, and align staffing where demand peaks. Researchers can declare SNT capture upfront and analyze it seriously. Payers can pilot risk adjustments or incentives where predictive value is proven. Each of these steps is incremental yet immediately actionable.

Forty years after Thompson—himself a nurse and the architect of the DRG system—first warned of its limitations, the need to rethink how we represent complexity in healthcare is more pressing than ever. Standardized nursing data are clinical data. They predict LOS. They anticipate transfer. They stratify survival. They sharpen equity. Integrating them into predictive and decision-making frameworks would finally bring their signal into the processes that govern care—not leave it unused. 

If we know these data can change outcomes, how much longer can we afford to silence them?
